# Protective Effects of D-Penicillamine on Catecholamine-Induced Myocardial Injury

**DOI:** 10.1155/2016/5213532

**Published:** 2015-12-14

**Authors:** Michal Říha, Pavlína Hašková, Jan Martin, Tomáš Filipský, Kateřina Váňová, Jaroslava Vávrová, Magdalena Holečková, Pavel Homola, Libor Vítek, Vladimír Palicka, Tomáš Šimůnek, Přemysl Mladěnka

**Affiliations:** ^1^Department of Pharmacology and Toxicology, Faculty of Pharmacy in Hradec Králové, Charles University in Prague, Heyrovského 1203, 500 05 Hradec Králové, Czech Republic; ^2^Department of Biochemical Sciences, Faculty of Pharmacy in Hradec Králové, Charles University in Prague, Heyrovského 1203, 500 05 Hradec Králové, Czech Republic; ^3^Department of Pharmacognosy, Faculty of Pharmacy in Hradec Králové, Charles University in Prague, Heyrovského 1203, 500 05 Hradec Králové, Czech Republic; ^4^Institute of Medical Biochemistry and Laboratory Diagnostics, 1st Faculty of Medicine, Charles University in Prague, Kateřinská 1660/32, 121 08 Prague, Czech Republic; ^5^Faculty of Medicine in Hradec Králové and University Hospital Hradec Králové, Charles University in Prague, Sokolská 581, 500 05 Hradec Králové, Czech Republic; ^6^4th Department of Internal Medicine, 1st Faculty of Medicine, Charles University in Prague, U Nemocnice 2, 128 02 Prague, Czech Republic

## Abstract

Iron and copper release participates in the myocardial injury under ischemic conditions and hence protection might be achieved by iron chelators. Data on copper chelation are, however, sparse. The effect of the clinically used copper chelator D-penicillamine in the catecholamine model of acute myocardial injury was tested in cardiomyoblast cell line H9c2 and in Wistar Han rats. D-Penicillamine had a protective effect against catecholamine-induced injury both* in vitro* and* in vivo*. It protected H9c2 cells against the catecholamine-induced viability loss in a dose-dependent manner. In animals, both intravenous D-penicillamine doses of 11 (low) and 44 mg/kg (high) decreased the mortality caused by s.c. isoprenaline (100 mg/kg) from 36% to 14% and 22%, respectively. However, whereas the low D-penicillamine dose decreased the release of cardiac troponin T (specific marker of myocardial injury), the high dose resulted in an increase. Interestingly, the high dose led to a marked elevation in plasma vitamin C. This might be related to potentiation of oxidative stress, as suggested by additional* in vitro* experiments with D-penicillamine (iron reduction and the Fenton reaction). In conclusion, D-penicillamine has protective potential against catecholamine-induced cardiotoxicity; however the optimal dose selection seems to be crucial for further application.

## 1. Introduction

Cardiovascular diseases remain the main cause of mortality in developed countries. The main culprit is atherosclerosis associated with coronary heart disease which can lead to its acute form, acute myocardial infarction [1]. The current treatment based mainly on the percutaneous coronary intervention has largely substituted other treatment modalities in developed countries. However, in some cases, due to contraindications or inaccessibility of adequate medical devices, particularly in developing countries, pharmacotherapy using fibrinolytics is still used. Irrespective of the procedure used, the recovery of blood flow of previously ischemic myocardium is associated with a phenomenon called reperfusion paradox [2]. This is based on a burst of reactive oxygen species (ROS) generated when previously ischemic tissue is reexposed to oxygenated blood flow. Release of free or loosely bound transition metals, namely, iron and copper, into the circulation plays an important role in this phenomenon, well documented in experimental animals [3, 4]. Our research group has previously shown that iron chelators might protect the myocardium from acute catecholamine-induced cardiac injury, which resembles the acute myocardial infarction in many aspects [5, 6]. Similarly, other groups have demonstrated some degree of protection by iron chelators in both experimental* in vivo* and clinical studies [7–9].

Much less attention has been paid to copper and its chelators and their potential effects in acute myocardial ischemic conditions. In human, copper elevation in patients with acute myocardial infarction has been well documented and a recent study has clearly demonstrated that higher serum copper is associated with higher cardiovascular mortality [10, 11]. To our knowledge, only one study tested the effect of a known copper chelator in an experimental model of myocardial ischemia. That study documented protective role of neocuproine against hydrogen peroxide induced cardiac damage and reperfusion arrhythmias in isolated perfused heart [12]. As far as we know, the effect of a copper chelator in a whole animal model of myocardial injury has never been tested. In the present study, we selected D-penicillamine (D-PA), a drug with a long history of clinical use in both copper-based and non-copper based pathologies. It is considered as the standard copper chelator and its rapid effect on copper excretion in urine is well documented [13, 14]. Although the drug is a close derivative of endogenous amino acid cysteine, it is known to have side effects (e.g., severe gastrointestinal disturbances, rash, proteinuria, and hematological adverse effects) when given in a long-term basis [15]. This is likely not true for its acute administration, where even the dose of 1.2 g/kg of D-PA did not produce toxic effects in rats [16]. For a possible clinical use of D-PA relevant to the acute myocardial injury, timely limited administration is suggested and therefore such therapy might be without significant adverse reactions. The main aim of this study was to assess if D-PA can protect cardiomyoblast derived cells and/or modify the acute myocardial injury caused by administration of synthetic catecholamine isoprenaline (ISO) in rats.

## 2. Materials and Methods

### 2.1. *In Vitro* Experiments

#### 2.1.1. Reagents and Solutions

**Figure 1:**
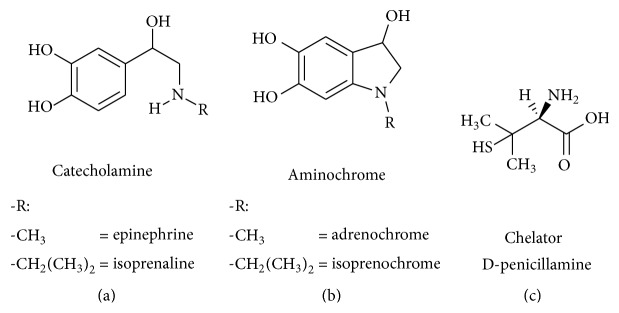
Chemical structures of compounds under investigation: (a) catecholamines epinephrine /(R)-4-(1-hydroxy-2-(methylamino)ethyl)benzene-1,2-diol/ and ISO /(RS)-4-(1-hydroxy-2-(isopropyl-amino)ethyl)benzene-1,2-diol/; (b) aminochromes, that is, catecholamine oxidation products, adrenochrome /3-hydroxy-1-methyl-2,3-dihydro-1H-indole-5,6-dione/ and isoprenochrome /3-hydro-xy-1-isopropyl-2,3-dihydro-1H-indole-5,6-dione/; and (c) copper chelator D-PA /(2S)-2-amino-3-methyl-3-sulfanyl-butanoic acid/.

The catecholamines epinephrine (EPI, Figure 1(a)) and ISO (Figure 1(a)), copper chelator D-PA (Figure 1(c)), ferric chloride hexahydrate (FeCl_3_·6H_2_O), ferrous sulphate heptahydrate (FeSO_4_·7H_2_O), 3-(2-pyridyl)-5,6-diphenyl-1,2,4-triazine-4′,4′′-disulphonic acid sodium salt (ferrozine), sodium acetate, acetic acid, 4-(2-hydroxyethyl)-1-piperazineethanesulphonic acid (HEPES), HEPES sodium salt, hydroxylamine, salicylic acid, and 2,3-dihydroxybenzoic and 2,5-dihydroxybenzoic acids were purchased from Sigma-Aldrich (USA). Deferoxamine was purchased from Novartis (Switzerland), dimethyl sulfoxide (DMSO) was purchased from Avantor Performance Materials (USA), and methanol for HPLC was purchased from JT Baker (USA). The chemicals and solutions used for cellular cultivation (cultivation media, sera, etc.) were purchased from Sigma-Aldrich or Lonza Group (Switzerland).

#### 2.1.2. Iron Chelation and Reduction Assay

Ferrozine is a specific indicator which forms a magenta colored complex with ferrous ions. In principle, ferric ions do not react with ferrozine. But the methodology can be extended for the assessment of total iron chelation after reduction of ferric ions by a suitable reductant like hydroxylamine. Similarly, iron reduction can be easily assessed. If a tested compound is able to reduce these ferric ions into ferrous ions, the indicator rapidly forms a complex with them which is thereafter measured spectrophotometrically [17].

Stock solutions of ferric ions and ferrous ions were prepared in distilled water (Milli-Q RG, Merck Millipore, USA). The corresponding fresh working solutions (0.25 mM) were prepared by dilution with distilled water. Hydroxylamine hydrochloride and ferrozine were dissolved in distilled water; D-PA was dissolved in DMSO. Experiments were performed in 15 mM buffers, acetate (pH 4.5 and 5.5) and HEPES (pH 6.8 and 7.5). Metal chelation experiments were performed in 96-well microplates, at least in duplicates, at room temperature using a Synergy HT Multi-Detection Microplate Reader (BioTek Instruments, Inc., USA) as described previously [17]. Briefly, for this assessment of iron chelation, various concentrations of D-PA were mixed in mentioned buffers with ferrous or ferric ions (at a final concentration of 25 *μ*M) for 2 min. In case of the assessment of total iron chelation, hydroxylamine was then added for reduction of nonchelated iron. The absorbance was measured immediately after the addition of ferrozine and after 5 min at 562 nm.

For the determination of the degree of ferric ions reduction, various concentrations of the tested compounds were mixed for 2 min with ferric ions in acetate or HEPES buffers. Afterwards, ferrozine was added and absorbance was measured at 562 nm immediately and after 5 min. Hydroxylamine was used as a positive control (100% reduction).

#### 2.1.3. Inhibition of Iron-Catalyzed Production of Hydroxyl Radicals

Ferrous ions react with hydrogen peroxide to produce hydroxyl radical (the Fenton reaction). The formed radical can be trapped by salicylic acid and its ensuing products (2,3-dihydroxybenzoic and 2,5-dihydroxybenzoic acids) can be detected by HPLC [18]. Briefly, ferrous ions were mixed with the tested compounds dissolved in methanol in different concentration ratios for 2 min. Salicylic acid and hydrogen peroxide (both 7 mM) were added subsequently, and the mixture was then analysed by HPLC (Dionex Ultimate 3000, Dionex Corp., USA) with Eclipse Plus C18 column (4.6 × 100 mm, 3.5 *μ*m, Agilent Inc., USA), using 40% methanol and 0.085% aqueous solution of phosphoric acid as a mobile phase. All experiments were checked by addition of “internal standard,” that is, known amounts of 2,3-dihydroxybenzoic and 2,5-dihydroxybenzoic acids.

#### 2.1.4. Cell Culture

The H9c2 cell line derived from embryonic BD1X rat heart tissue [19] was obtained from the American Type Culture Collection (ATCC, USA). Cells were cultured in Dulbecco's modified Eagle's medium (DMEM; Lonza) supplemented with 10% heat-inactivated fetal bovine serum (FBS; Lonza), 1% penicillin/streptomycin solution (Lonza), and 10 mM HEPES buffer (pH 7.4; Sigma). Cell cultivation was performed in 75 cm^2^ tissue culture flasks from Techno Plastic Products AG (TPP) at 37°C in a humidified atmosphere of 5% CO_2_ in air. Cells were subcultured twice in a week when they reached approximately 90% confluence (i.e., every 3rd-4th day).

For particular experiments, cells were seeded into appropriate microplates (TPP) at given cellular density. The medium was changed for serum-free cell-culture medium (pyruvate-free DMEM (Sigma) supplemented with 1% penicillin/streptomycin solution (Lonza) and 10 mM HEPES buffer (pH 7.4; Sigma)) 24 h prior to all cellular experiments. Serum deprivation was used to stop cellular proliferation to mimic the situation in postmitotic cardiomyocytes [20]. Pyruvate was omitted because it is an antioxidant and may interfere with ROS-related toxicity. The lipophilic compounds were dissolved in DMSO, yielding a final concentration of 0.1% in all experimental groups. At this concentration, DMSO had no effect on cellular viability.

#### 2.1.5. Cell Experiments

**Figure 2:**
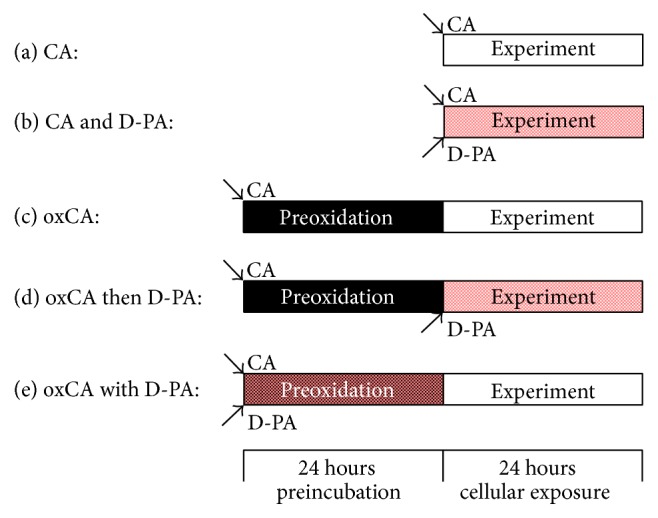
Overview of the protocols for cell experiments using the H9c2 cardiomyoblast cell line. Catecholamines (CA-ISO or epinephrine) were added to H9c2 cells either as freshly prepared solutions (a and b) or after 24 h preincubation in the cell-culture medium at 37 °C (oxidized oxCA) (c–e). D-PA was added either at the beginning of 24 h cellular experiments (b and d) or at the start of 24 h catecholamine preincubation before the 24 h cellular experiments (e).

All cell experiments were based on the ability of catecholamines to undergo spontaneous oxidation to a number of chemically related products [21]. For that reason, there were created five different protocols for the study of toxic and protective properties of compounds under investigation (Figure 2). In brief, work solutions of catecholamine (ISO or EPI) used in all experiments were either freshly prepared or 24 h preoxidized (i.e., left spontaneously to oxidize) at 37°C. The copper chelator D-PA was added into the solution with catecholamine either at the beginning of the cell experiment itself or at the beginning of 24 h preoxidaton of catecholamine.

#### 2.1.6. Neutral Red Uptake Assay for Assessment of Compounds Cytotoxicity

Cellular viability was determined using the assay based on the ability of viable cells to incorporate neutral red (NR; Sigma). This well-established assay consists in the readily penetration of intact cell membranes by weak cationic dye, NR, and its accumulation in the lysosomes of viable cells [22]. H9c2 cells seeded in 96-well plates at a density of 10,000 cells per well were incubated with compounds under investigation (alone or in combinations) for 24 h. Half of the medium volume from each well was removed at the end of incubation, and the same volume of medium with NR was added, yielding a final concentration of 40 *μ*g/mL. After 3 h at 37°C, the supernatant was discarded, and the cells were fixed in 0.5% formaldehyde supplemented with 1% CaCl_2_ for 15 min. The cells were then washed twice with phosphate buffered saline and solubilized with 1% acetic acid in 50% ethanol for 30 min of continuous agitation; thus the accumulated NR was released into the extracellular fluid. The light absorption (optical density) of released NR was measured using a microplate spectrophotometer Tecan Infinite 200 M (Tecan, Switzerland) at *λ* = 540 nm. The viability of experimental groups was expressed as a percentage of the untreated control (100%).

#### 2.1.7. Epifluorescence Microscopy for Imaging of Cellular Morphology Changes

Changes in cellular morphology were observed and imaged using an inverted epifluorescence microscope Nikon Eclipse TS100 with 10–40x air objectives (Nikon, Japan) equipped with a digital camera 1300Q (VDS Vosskühler GmbH, Germany) and the software NIS-Elements AR 3.0 (Laboratory Imaging s.r.o., Czech Republic). The cellular viability was visualized using nuclei staining with Hoechst 33342 (Molecular Probes, USA) and propidium iodide (PI; Molecular Probes), which are well-established and sensitive probes to determine apoptosis and necrosis: Hoechst 33342 is a blue-fluorescent probe (*λ*_ex_ = 360 nm, *λ*_em_ = 460 nm) staining all nuclei. In apoptotic cells, chromatin condensation occurs and apoptotic cells can thus be identified as those with condensed and more intensely stained chromatin. The red-fluorescent (*λ*_ex_ = 560 nm, *λ*_em_ = 630 nm) DNA-binding dye, PI, is unable to cross the plasma membrane of living cells but readily enters necrotic (or late-stage apoptotic) cells and stains their nuclei red. H9c2 cells seeded in 12-well plates at a density of 75,000 cells per well were incubated with compounds under investigation (alone or in combinations) for 24 h. After that, cells were stained with 10 *μ*g/mL Hoechst 33342 and 1 *μ*g/mL PI for 10 min at 37°C.

### 2.2. *In Vivo* Experiments

#### 2.2.1. Animals

Forty-six Wistar Han male rats were obtained from Meditox (Czech Republic) and housed in cages located in a special air-conditioned room with a periodic light-dark (12-12 h) cycle for 2 weeks. During this period, they were provided with free access to tap water and standard pellet diet for rodents. After the acclimatization period, healthy rats weighing approximately 390 g were used for the experiments.

The study was approved by the Experimental Animal Welfare Committee of Charles University in Prague, Faculty of Pharmacy in Hradec Králové, and conformed to the Guide for the Care and Use of Laboratory Animals published by the US National Institutes of Health (NIH Publication Number 85-23, revised 1996).

#### 2.2.2. Experimental Design

The rats were randomly divided into six groups. Firstly, D-PA in the doses of 11 or 44 mg/kg or water for injection (B. Braun, Germany, 2 mL/kg) was administered into the tail vein. ISO (Sigma-Aldrich, 100 mg/kg, 50 mg/mL) or water for injection was given s.c. 5 min later. The groups in which rats received ISO are designated as ISO (positive control), D-PA11+ISO, and D-PA44+ISO, while the others are designated as C (negative control), D-PA11, and D-PA44.

#### 2.2.3. Anaesthesia and Surgery

The animals had free access to water and diet during the first 12 h after drug administration. The rats were then fasted for the next 12 h before the surgery. Animals were anaesthetized with i.p. injection containing aqueous solution of urethane (Sigma-Aldrich) in a dose of 1.2 g/kg. Surgical and instrumental procedures were similar to our previous studies [6]. Briefly, the left common iliac artery was connected to a pressure transducer MLT0380/D (ADInstruments, Australia)* via* a polyethylene catheter (0.5/1.0 mm filled with heparinized saline 50 IU/mL) for arterial blood pressure measurement. A high-fidelity pressure-volume micromanometer catheter (Millar pressure-volume catheter SPR-838 2 F, 4E, 9 mm, Millar Instruments Inc., USA) was inserted into the left heart ventricle through the right common carotid artery. Both pressure transducer and Millar pressure-volume catheter together with subcutaneous electrodes for the ECG standard limb lead II MLA1215 (ADInstruments) were connected to PowerLab with LabChart 7 software (ADInstruments). Data were analyzed for 30 min, and necessary calibrations with hypertonic saline (2 × 20 *μ*L of 25% w/w sodium chloride solution) were performed at the end of the experiment. A blood sample was collected from the abdominal aorta into a heparinized test tube (170 IU). Following the experiment, all surviving animals were killed painlessly in anaesthesia by intravenous administration of 1 mL of 1 M aqueous solution of potassium chloride (Sigma-Aldrich).

#### 2.2.4. Biochemical Analyses

Cardiac troponin T (cTnT) was measured in serum; vitamin C and vitamin E were measured in plasma. cTnT was determined by high sensitive electrochemiluminescence immunoassay (Cobas e411, Roche) using two monoclonal antibodies specifically directed against cTnT. Vitamin E was measured with fluorometric detection after deproteinization in an HPLC system LC-10A (Shimadzu, Japan). Analogously, vitamin C was measured after deproteinization by electrophoresis using UV detection (PrinCE 750, Netherlands).

For lipid peroxidation measurement, 100–150 mg of heart tissue was diluted 1 : 9 (by weight) in ice-cold 0.1 M potassium phosphate buffer. The tissue was diced and then sonicated with ultrasonic cell disruptor (Model XL2000, Misonics, USA). 20 *μ*L of heart sonicate was incubated for 30 min in 37°C with 100 *μ*M ascorbate (Sigma-Aldrich) and 6 *μ*M FeSO_4_ as previously described [23]. The amount of CO produced into vial was quantified by gas chromatography with reduction gas detector serving as an index of lipid peroxidation.

### 2.3. Data Analysis

The amount of nonchelated or reduced iron was calculated from the difference of absorbance between the tested sample (with ferrozine) and its corresponding blank (without ferrozine) divided by the difference of the control sample (the known amount of iron without the tested substance) and its control blank. The concentration of hydroxyl radical was calculated as the mean of the samples mixed (1) with methanol and (2) with added internal standards. Calculations in animal study were performed as previously described [6].

Grubb's test was used for detection of outlier values in animal and cell culture studies. Data were expressed as mean ± SD. For multiple comparisons, one-way ANOVA followed by Tukey's multiple comparisons test (*in vivo* experiments) and Bonferroni* post hoc* analysis (cell culture study) were used. Differences between groups were considered significant at *P* < 0.05 unless indicated otherwise. All statistical analyses were performed by GraphPad Prism version 6.0 for Windows (GraphPad Software, USA) except for the cell culture experiments where the statistical software SigmaStat for Windows 3.5 (Systat Software, Inc., USA) was used.

## 3. Results

### 3.1. *In Vitro* Experiments

**Figure 3:**
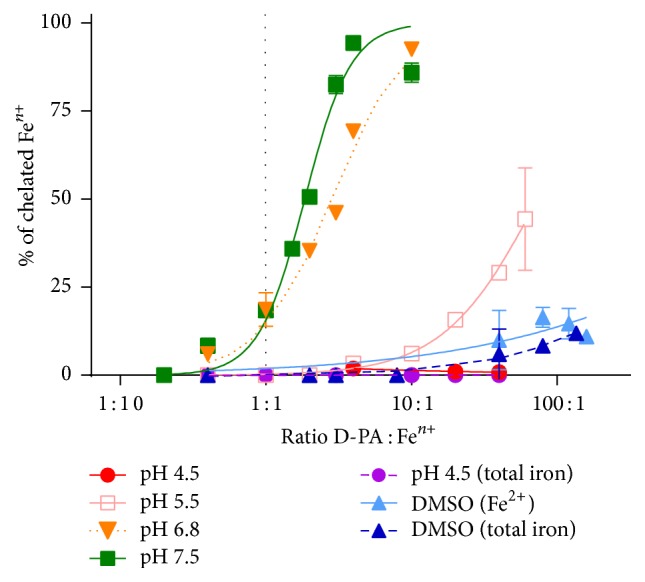
Iron-chelating activity of D-PA at (patho)physiologically relevant pH conditions and nonbuffered conditions (DMSO).

Since metal chelators are generally not very specific which seems to be true for D-PA as well [24, 25], we were firstly interested if D-PA can chelate iron. Our competitive spectrophotometric approach confirmed the ability of D-PA to chelate iron. However, the chelating capacity dropped with decreasing pH (Figure 3).

**Figure 4:**
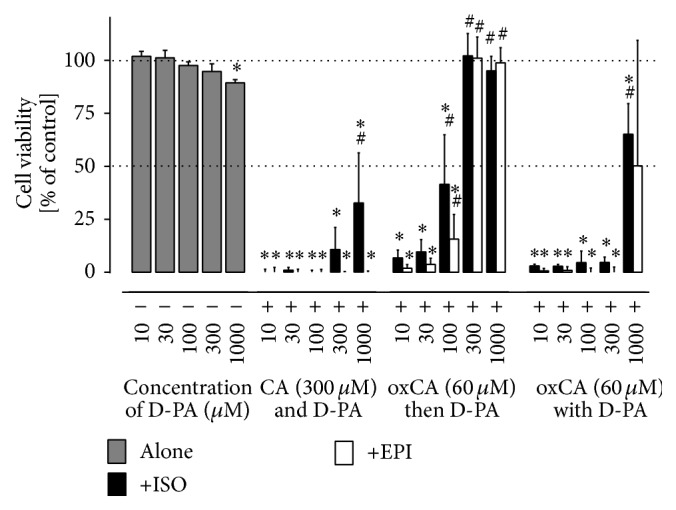
Cell viability studies using the H9c2 cardiomyoblast cell line. Figure shows dose-dependent own toxicity of D-PA (10–1000 *μ*M,* left*) and its protective effects against toxicity induced by catecholamines (CA) ISO and EPI. D-PA was added to freshly prepared catecholamine solution before the start of 24 h cell experiments (CA and D-PA,* middle left*); D-PA was added immediately before cellular experiments to catecholamines preincubated for 24 h in cell-culture medium (oxCA then D-PA,* middle right*); or D-PA was preincubated for 24 h together with catecholamines and then added to cells (oxCA with D-PA,* right*). Schematic overview of experimental protocols is in Figure 2. Cell viability was determined by neutral red uptake assay and expressed as a percentage of the untreated control group. Data are presented as means ± SD; *n* = 4; statistical significance (ANOVA) ^*∗*^ *P* < 0.05 versus control and ^#^ *P* < 0.05 versus corresponding catecholamine group.

As we have previously shown, compounds with iron-chelating potential could reduce catecholamine cardiotoxicity both* in vitro* and* in vivo* [5]; we firstly assessed the effect of D-PA on cell damage caused by catecholamines EPI and ISO and their oxidation products (oxEPI and oxISO) in cardiomyoblast H9c2 cell line (Figure 4). D-PA alone did not significantly influence the cell viability in the tested concentration range (10–1000 *μ*M) which is achievable in plasma by administration of D-PA [26]. However, D-PA was able to restore the viability after catecholamine treatment in a dose-dependent manner. Interestingly, D-PA mediated protection was observed especially in cells treated with preoxidated catecholamines. In this case, 300 and 1000 *μ*M of D-PA completely prevented cell death.

Changes in cell morphology were observed and imaged using an inverted epifluorescence microscope in H9c2 cardiomyoblasts (Figure S1 in Supplementary Material available online at http://dx.doi.org/10.1155/2016/5213532). While necrotic (or late-stage apoptotic) cells were found after 24 h preincubated catecholamines ISO and EPI (ox-ISO and ox-EPI, resp.), 300 *μ*M D-PA markedly prevented the damage of cardiomyoblasts. D-PA alone did not apparently change cellular morphology.

### 3.2. *In Vivo* Study

**Figure 5:**
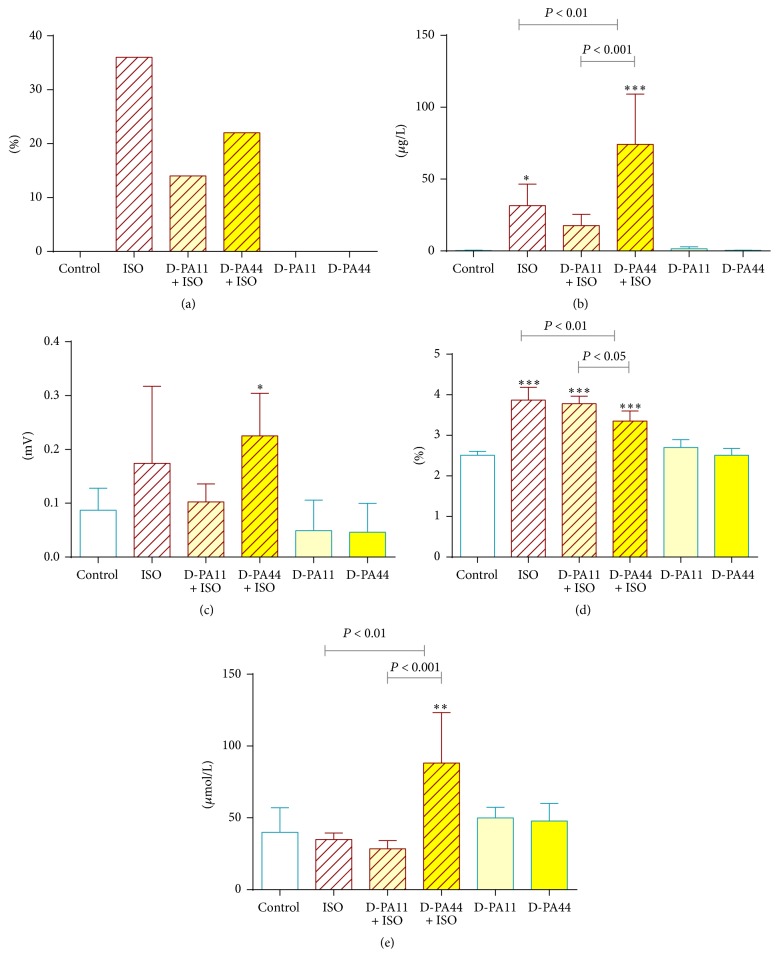
Mortality (a), changes in levels of cardiac troponin T (b), QRS-T junction (c), wet ventricles weight index (d), and levels of vitamin C in plasma (e). Statistical significance versus control: ^*∗*^ *P* < 0.05, ^*∗∗*^ *P* < 0.01, and ^*∗∗∗*^ *P* < 0.001.

With regard to the protective action of D-PA* in vitro*, its* in vivo* effects on rats were consecutively tested. The s.c. ISO dose of 100 mg/kg (after i.v. dose of solvent) caused 4 deaths of 11 rats (36% mortality) within 24 h. Intravenous premedication by two doses of D-PA (11 and 44 mg/kg) decreased the mortality in ISO-treated animals in both doses (Figure 5(a)). The lower dose of D-PA was more beneficial in comparison with the higher one (14% versus 22%, 1 death of 7 rats and 2 deaths of 9 rats, resp.). Four rats (1 in the ISO group, 2 in D-PA44+ISO, and 1 in D-PA44 group) died during the surgery. These deaths, however, were likely not to be linked to the type of treatment but were caused by problems during the surgical procedure. Except for one animal in D-PA44 group, mentioned above, no deaths occurred in any control rat which did not receive ISO.

The lower mortality in the group with the lower D-PA dose in comparison to positive ISO group apparently corresponded to a lower release of cTnT (Figure 5(b)). There was not a significant difference in cTnT levels between D-PA11+ISO and ISO. However, there was also no difference between D-PA11+ISO and controls suggesting a partial protection by this lower dose. Unexpectedly, higher dose of D-PA with ISO increased significantly cTnT when compared to both the controls and the ISO group. Only very low serum levels of cTnT were found in all rats which did not receive ISO. Data on QRS-T junction (analogous to ST-segment elevation in human) corresponded to cTnT results (Figure 5(c)). An increase of wet ventricles weight index after administration of ISO indicates myocardial oedema in these acute experiments [27]. The effects of both lower and higher dose of D-PA on wet ventricles weight index (Figure 5(d)) were opposite to our above-mentioned results. The higher dose of D-PA improved myocardial oedema, while the lower dose only tended to improve it.

*Hemodynamic Parameters*. ISO treatment did not significantly modify blood pressure, but it accelerated heart rate and decreased stroke volume and ejection fraction 24 h after drug administration. D-PA alone in both doses did not affect the mentioned parameters in comparison to the solvent control group. Coadministration of ISO with D-PA did not lead to the normalization of the hemodynamic parameters with exception of the higher dose, where the drop in stroke volume was prevented. Representative hemodynamic parameters are depicted in Figure S2.

Nonsignificant changes were observed in other hemodynamic parameters, especially left-ventricular end-diastolic pressure, developed pressure, *dp*/*dt*_max_, and *dp*/*dt*_min_. The relaxation parameter (the time constant of left ventricular isovolumic pressure decay, tau) was likely due to high variability only insignificantly elevated in the ISO group in comparison to controls (data not shown).

*Markers of Oxidative Stress*. The level of vitamin C in plasma in both D-PA groups and solvent were almost identical and only insignificant drop was observed in ISO group which was analogous in the case of D-PA11+ISO group. However, the higher dose of D-PA markedly increased serum concentration of vitamin C in ISO-treated rats (Figure 5(e)). No changes in serum concentration of vitamin E and lipid peroxidation in the heart were observed (data not shown).

### 3.3. Additional* In Vitro* Experiments

**Figure 6:**
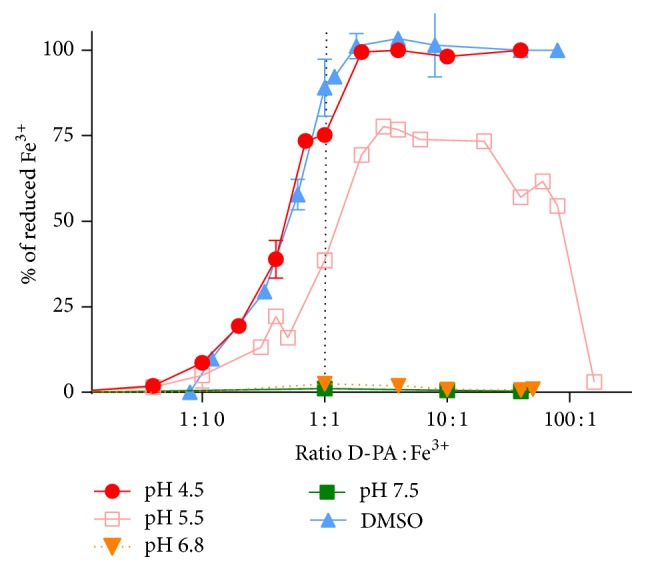
Reduction of ferric ions by D-PA at four pH conditions and at nonbuffered conditions (DMSO).

Since the results of lower and higher dose of D-PA were divergent in the* in vivo* study and, in particular, the higher dose of D-PA markedly increased levels of vitamin C in plasma, additional* in vitro* experiments were performed in order to assess possible anti- or prooxidant action of D-PA. D-PA substantially reduced ferric ions and this reduction was dependent on the acidity of the environment, especially at pH 4.5 or at nonbuffered conditions; iron was completely reduced from the ratio 2 : 1 (D-PA : Fe^3+^, Figure 6).

**Figure 7:**
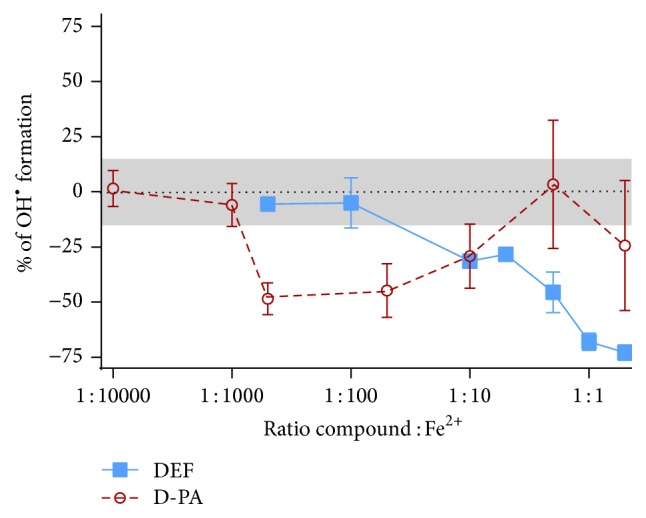
The impact of D-PA and the standard iron chelator deferoxamine (DEF) on hydroxyl radical formation. Grey area represents the error of the method.

Since iron reduction may lead to potentiation of the Fenton chemistry, we also tested the influence of D-PA on the* in vitro* production of hydroxyl radical in the iron-catalyzed Fenton reaction using the HPLC system (Figure 7). In contrast to the standard iron chelator deferoxamine (DEF) which dose-dependently decreased the formation of hydroxyl radical, D-PA showed more complex, inverse bell-shaped behaviour. At very low ratios of D-PA to Fe^2+^, D-PA efficiently blocked the Fenton reaction, while at higher ratios the effect of this amine was neutral.

## 4. Discussion

D-PA is a drug with complex mechanism of action as can be documented by its large therapeutic indication. D-PA represents the basic decoppering agent used for the treatment of Wilson's disease [28]. Other indications include especially rheumatoid arthritis, cystinuria, scleroderma, or heavy metal poisoning [29]. Our first idea was to test this drug mainly because of its known effect on copper homeostasis. Although its copper chelation effect may be limited [14, 30], it is well known for its ability to induce copper excretion in the urine [13, 26]. Therefore, the main hypothesis was that copper released from ischemic myocardium could be chelated or mobilized to urine by D-PA, which might protect myocardium from catecholamine-induced injury. Furthermore, since iron is also known to be released during myocardial ischemia [3, 4] and metal chelators including D-PA are generally not completely selective, we also evaluated D-PA chelating effect on iron ions. Interestingly, D-PA showed pronounced and stable iron chelation effects at neutral or slightly acidic pH in our experiments (Figure 3). While more than 80% of ferrous ions were chelated at the concentration ratio of 10 : 1 (D-PA : Fe^2+^) at slightly acidic conditions or physiological pH, neither cuprous nor cupric ions were bound by D-PA at the same ratio [30]. This was rather unexpected since a previous report has shown that the affinity of D-PA for iron is not particularly high and lower than that for other metals [31]. However, chelation and/or increased excretion of both iron and copper might be positive for the use of D-PA in the case of ROS-mediated myocardial injury. Moreover, before we started to test D-PA in rats, we firstly tested its effects on cardiomyoblast cells. D-PA had apparently very low toxicity and clear protection against catecholamines was seen at this level (Figures 4 and S1). Our previous experiments with a strong and lipophilic iron chelator salicylaldehyde isonicotinoyl hydrazone (SIH) revealed that the protective action of chelation with SIH was associated with at least two distinct effects: (i) slowing down the progressive catecholamine oxidation to reactive toxic intermediates and (ii) reduction of toxicity of the already formed oxidation products [21]. The present results suggest that D-PA may act by similar mechanisms.

The injury caused by synthetic catecholamine ISO resembles in many aspects the acute myocardial infarction in human. For an* in vivo* model of myocardial infarction, we administered ISO in a dose of 100 mg/kg s.c. which is a generally used dosage to produce significant mortality with about 1/3 of deceased animals within 24 h [5, 6, 32, 33]. The mortality in our current study was analogous (36%). In agreement with* in vitro* findings, both doses of D-PA reduced the mortality but the effect was not dose-dependent (lower dose decreased the mortality to 14% while the higher to 22%). In our previous experiments, only two iron-chelating compounds had clearly protecting effects on the mentioned model in the terms of the most severe parameter, the mortality: 2-pyridylcarboxaldehyde 2-thiophenecarboxyl hydrazone in the dose of 20 mg/kg completely prevented the mortality, while the lower dose of 10 mg/kg did not have an effect [5] and dexrazoxane in the dose of 20.4 mg/kg reduced the mortality to 12.5% [6]. Other drugs with iron-chelating properties, namely, deferoxamine and lactoferrin, were without any effect, while rutin deteriorated the mortality [5, 32, 33].

The results with D-PA were different since both lower and higher doses of D-PA evoked apparently diverse effects on our model. The lower dose, 11 mg/kg, is equimolar to 50 mg/kg of deferoxamine and was selected for reason of comparison on the molar basis with other chelators as in our previous studies [5]. Moreover, this dose fits in the dosage range in the treatment of Wilson's disease [28]. Higher dose was added to the study protocol because of partially protective effects of the lower dose. Both doses decreased the mortality, but the mechanism does not seem to be identical. The lower dose decreased the release of cTnT suggesting cardioprotection, while the higher dose had rather an opposite effect on cTnT but decreased the wet ventricles weight index and normalized the stroke volume. From the biochemical point of view, it is quite surprising that the higher dose of D-PA evoked the marked increase in plasma vitamin C (Figure 5(e)). It is not easy to explain this result which was found only in the combination of D-PA with isoprenaline but not with the solvent. It is therefore likely linked to the toxic effects of isoprenaline. Oxidative stress might play a role. We are not the first to suggest it, since (1) hydrogen peroxide production from D-PA after the addition of copper was documented [34, 35], (2) D-PA in high doses produces intracellular oxidative stress in cell experiments but antioxidant enzymes might be both upregulated or downregulated [36], and (3) a recent clinical study showed that treatment with D-PA led to significant decrease in the activity of glutathione peroxidase, one of the most important antioxidant enzymes in blood, and a tendency to increase the total antioxidant capacity [37]. The latter is a similar finding to the significant plasma vitamin C increase reported in the present study. On the other hand, it should be mentioned that, in healthy animals, D-PA in these acute settings was very well tolerated in both doses and we did not observe any negative haemodynamic changes. Therefore, as suggested above, the negative effect of the higher dose of D-PA seems to be related to ischemia caused by administration of ISO or to direct effect of this catecholamine and/or its oxidation products. Ischemia leads to a decrease in pH [38] and one of the plausible mechanisms of D-PA prooxidation is iron reduction seen particularly at lower pH (Figure 6). In addition, copper reduction by D-PA was documented in our previous study [30]. It is well known that reduction of iron or copper intensifies the Fenton reaction due to redox-cycling of the catalyst [39] and therefore D-PA could increase the production of hydroxyl radical. D-PA behaviour in relation to the Fenton chemistry appears to be similar to some reducing antioxidants [40], since D-PA acted as an efficient antioxidant in low concentrations but its protective properties decreased or were even reversed at higher ratios (Figure 7). This may explain the unexpected effect of the higher dose of D-PA in the animal model but we are aware that it is not possible to directly transform* in vitro* data into* in vivo* situation. Although it is tempting to speculate that increased oxidative stress could explain why the higher dose was not more efficient than the lower one, more* in vivo* experiments will be needed to confirm it. On the other hand, the high dose of D-PA had some protective effects which were very different from those of the lower dose and might be caused by other mechanisms apart from interaction with transition metals. Since D-PA had some effect in autoimmune disease, especially rheumatoid arthritis and scleroderma [29], one could speculate that D-PA can inhibit the activation of the immune system [35, 41], which is an important component of the myocardial injury [42]. Indeed, wet ventricle weight might increase due to myocardial oedema caused by activation of immune system [27] and this parameter was decreased by the higher dose of D-PA. However, the direct effect of D-PA on immune system in acute myocardial injury was not tested in this study.

## 5. Conclusion

In conclusion, this study has shown that D-PA has potential cardioprotective effects on acute myocardial injury caused by catecholamines likely due to its effect(s) on copper and/or iron homeostasis. However, in higher doses, despite positive effects on some cardiovascular parameters (normalization of stroke volume and decrease of wet ventricles weight index), the overall protective effect was attenuated, possibly due to the reduction of transition metals followed by prooxidation. The mentioned positive hemodynamic effects were presented only in the higher dose and do not seem to be based on interaction with transition metals.

## Supplementary Material

Figure S1: Cell morphology and nuclear epifluorescence co-staining with Hoechst 33342 and propidium iodide of H9c2 cardiomyoblasts.
Figure S2: The effect of D-PA on haemodynamic parameters: mean blood pressure (a), heart rate (b), stroke volume (c) and ejection fraction (d). Statistical significance vs control: ∗ p < 0.05, ∗∗ p < 0.01 vs control.

## Abbreviations

CA: Catecholamine
cTnT: Cardiac troponin T
D-PA: D-Penicillamine
EPI: Epinephrine
ISO: Isoprenaline
NR: Neutral red
PI: Propidium iodide
ROS: Reactive oxygen species
SIH: Salicylaldehyde isonicotinoyl hydrazone.
