# Supplementary material for: Protective Effects of D-Penicillamine on Catecholamine-Induced Myocardial Injury
Source: Oxid Med Cell Longev. 2015 Dec 14;2016:5213532. doi: 10.1155/2016/5213532 (PMC4691625; doi:10.1155/2016/5213532)
Supplement: Supplementary file 1 — Figure S1: Cell morphology and nuclear epifluorescence co-staining with Hoechst 33342 and propidium iodide of H9c2 cardiomyoblasts. Figure S2: The effect of D-PA on haemodynamic parameters: mean blood pressure (a), heart rate (b), stroke volume (c) and ejection fraction (d). Statistical significance vs control: ∗ p < 0.05, ∗∗ p < 0.01 vs control. [file 5213532.f1.doc]

[
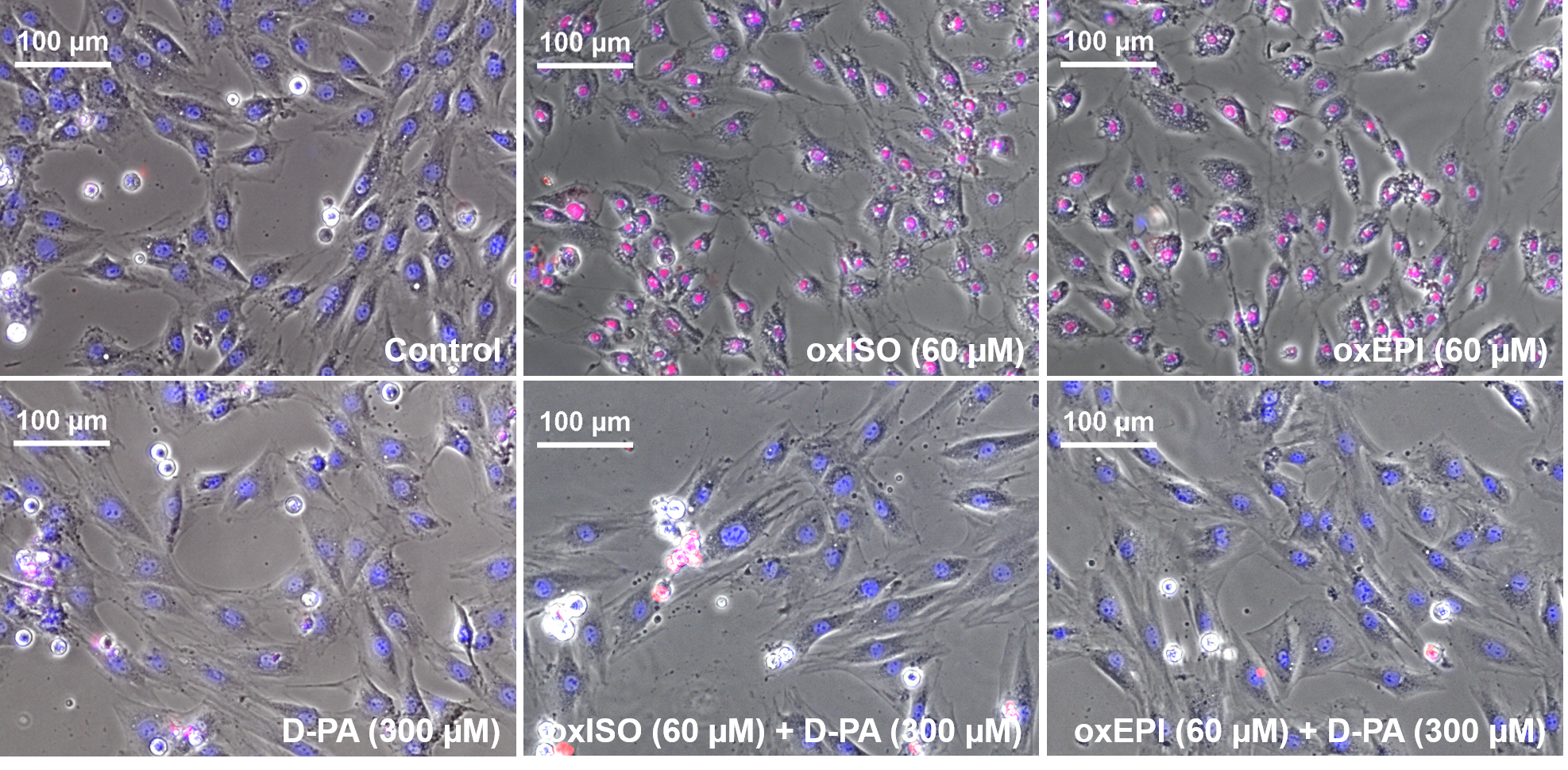
](mailto:mladenkap@faf.cuni.cz%0C%01)

**Figure S1: Cell morphology and nuclear epifluorescence co-staining with Hoechst 33342 and propidium iodide of H9c2 cardiomyoblasts.** Cells were incubated for 24 h with control medium; with a 24h-preincubated catecholamine (oxISO or oxEPI); and with the copper chelator D-PA; or with their combination, when D-PA was added immediately before cellular experiments to catecholamines preincubated for 24 h in cell-culture medium (oxCA then D-PA). The blue-fluorescent probe Hoechst 33342 stained all nuclei of H9c2 cells; the red-fluorescent dye PI stained nuclei only of necrotic (or late-stage apoptotic) cells. Scale bars represent 100 µm. Images are typical of four experiments.


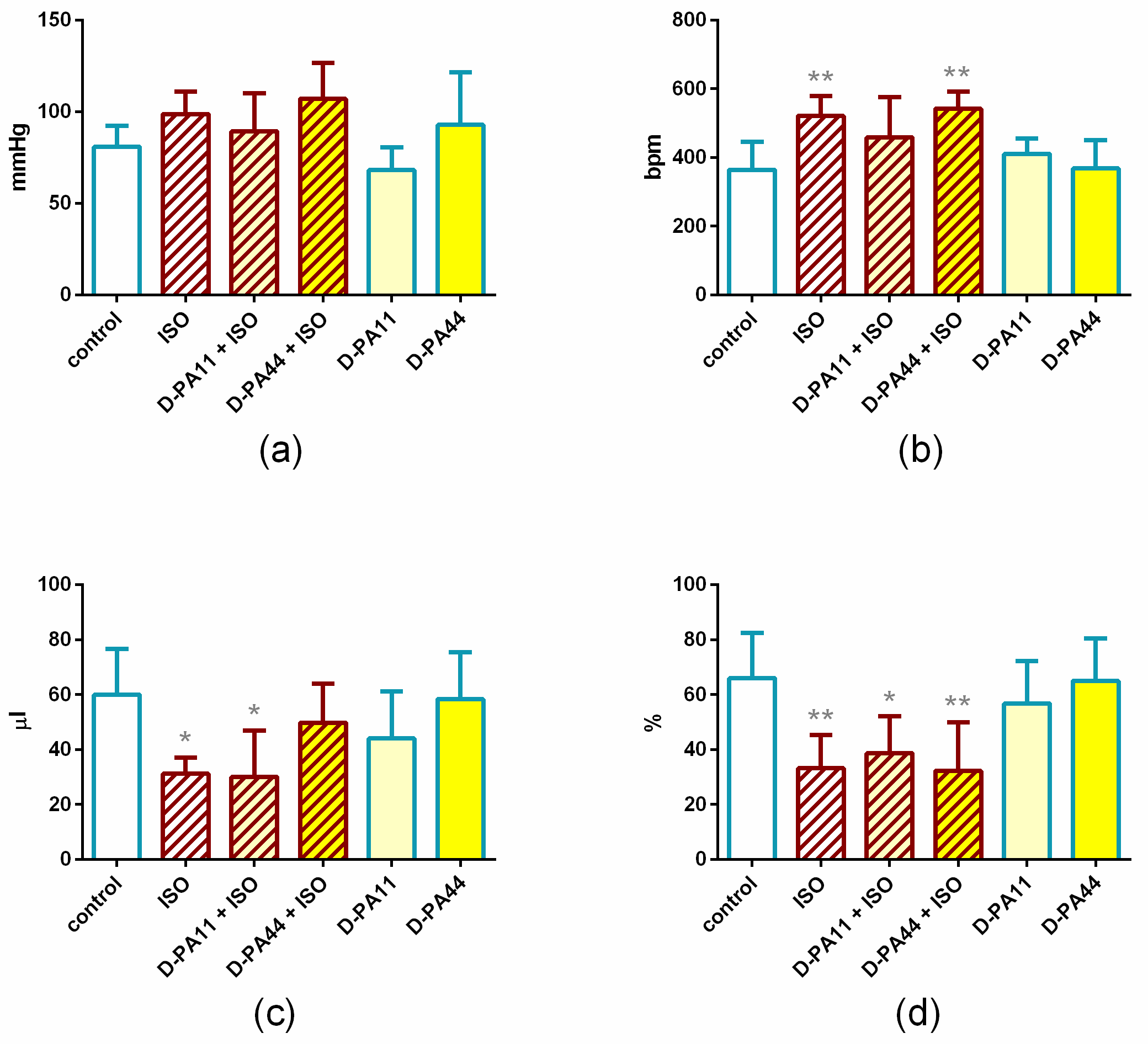


FIGURE S2: The effect of D-PA on haemodynamic parameters: mean blood pressure (a), heart rate (b), stroke volume (c) and ejection fraction (d). Statistical significance vs control: * p < 0.05, ** p < 0.01 vs control
